# Alterations in regional right ventricular shape in patients following tetralogy of Fallot repair involving a transannular Patch

**DOI:** 10.1186/1532-429X-18-S1-P182

**Published:** 2016-01-27

**Authors:** Amita Singh, Anuj Mediratta, Francesco Maffessanti, Shelby Kutty, Victor Mor-Avi, Roberto Lang, Amit R Patel

**Affiliations:** 1 grid.412578.d0000000087369513Cardiology, University of Chicago Hospitals, Chicago, IL USA; 2 grid.266813.80000000106664105Cardiology, University of Nebraska, Omaha, NE USA

## Background

Patients who undergo surgical repair of Tetralogy of Fallot (TOF) often experience abnormal remodeling of the right ventricle (RV), notably with chamber dilatation and associated dysfunction. The ideal surgical technique is controversial, but RV outflow tract repair utilizing a transannular patch (TAP) is employed to overcome the RV outflow tract obstruction in many patients with TOF. We hypothesized that analysis of both RV volumes and curvature in the different regions of the right ventricle can unmask differences in RV remodeling patterns following repair using these two surgical approaches.

## Methods

Cardiovascular magnetic resonance (CMR) was performed in 25 patients with repaired TOF. End-diastolic and end-systolic endocardial surfaces were manually segmented from a short-axis cine stack, coronal and 4-chamber views of the RV to measure end-diastolic volume (RVEDV), end-systolic volume (RVESV), and ejection fraction (RVEF). A 3D model of the RV was generated from multiple short and long axis images using custom software (CMR^2^V in MATLAB) that measures regional curvature in RV segments (RV inflow, RV outflow, free wall and septum).

## Results

**Figure 1:**
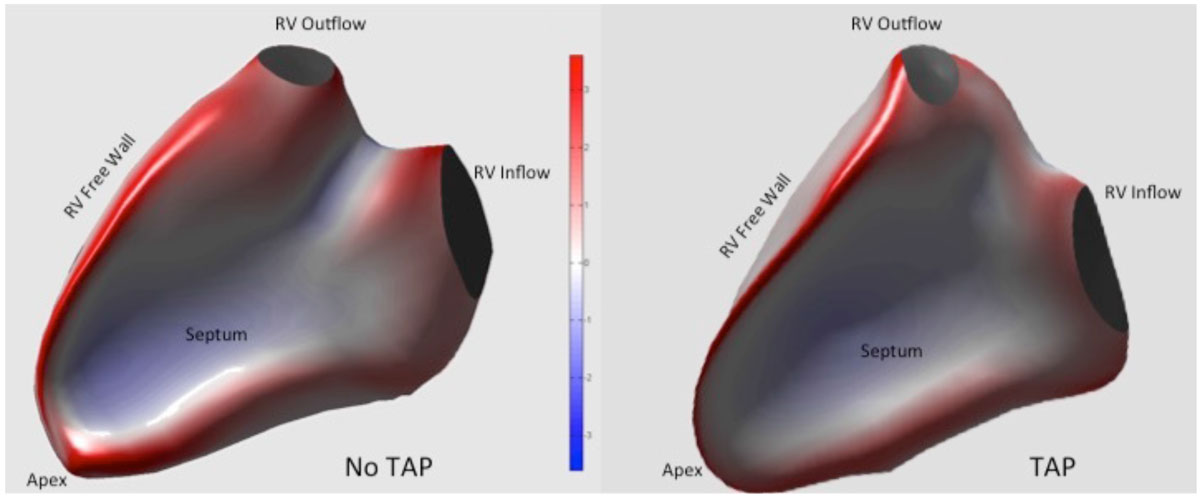
Figure 1

Eleven patients (44%) had a repair involving a TAP (mean age 13.9 ± 4.3 years; 88% male), and 14 patients had no TAP (mean age 16.5 ± 13.3 years; 43% male). Both groups had similar reductions in RVEF and increases in RVEDV and RVESV (Table 1). Regional curvature differed significantly between groups in the mid free wall and in the free wall adjacent to the RVOT region, with TAP patients depicting higher curvature (more convex, directed outward from the RV) compared to patients without TAP. The mid septal region curvature was lower (more concave, and relatively flatter) in patients with TAP versus those without (see Figure 1).

**Table 1:** RV Volume and Geometric Parameters

	EDVi (mL/m2)	ESVi (mL/m2)	RV EF(%)	Septum Curvature	Free Wall Curvature	RVOT/Free Wall Curvature
TAP (n = 11)	138 ± 15	85 ± 16	38 ± 11	0.61 ± .07 *	1.54 ± .06 *	1.38 ± .09*
No TAP (n = 14)	128 ± 31	86 ± 29	34 ± 9	0.71 ± .08	1.45 ± .09	1.26 ± .09

TAP = Transannular patch; EF = ejection fraction; EDVi = End diastolic volume index; ESVi = End systolic volume index; RVOT = Right ventricular outflow tract

* p-value <0.05 between groups

## Conclusions

In patients with repaired TOF with TAP repair compared to non-TAP repair, RV shape analysis demonstrated differences in regional geometry and RV curvature, despite similar intergroup volumetric and functional indices. Segmental regional curvature in operated TOF patients may be useful to provide a better understanding of the RV remodeling process after TOF repair using these different surgical approaches.

